# Analysis of ischemic stroke burden in Asia from 1990 to 2019: based on the global burden of disease 2019 data

**DOI:** 10.3389/fneur.2023.1309931

**Published:** 2023-12-22

**Authors:** Xueli Zhang, Hengliang Lv, Xin Chen, Maoxuan Li, Xiaojing Zhou, Xiaoying Jia

**Affiliations:** ^1^School of Health Management, Changchun University of Chinese Medicine, Changchun, China; ^2^Jilin Provincial People’s Hospital, Changchun, China; ^3^Department of Epidemiology, School of Public Health, China Medical University, Shenyang, China; ^4^School of Public Health, Nanjing Medical University, Nanjing, China; ^5^Department of Biochemistry, Clinical Medical College, Changchun University of Chinese Medicine, Changchun, China

**Keywords:** ischemic stroke, Asia, burden, temporal, region, population

## Abstract

**Background:**

Ischemic stroke has the characteristics of high morbidity, mortality, and recurrence rate. This study aimed to describe and assess the burden of ischemic stroke in Asia based on the global burden of disease (GBD) 2019 data and provide a crucial scientific foundation for the prevention and control of this life-threatening disease.

**Method:**

This study used the GBD 2019 data to assess the burden of ischemic stroke. The indicators used in this study were derived from the following methods: (i) the incidence of ischemic stroke was estimated using the disability model for the global burden of disease study-mixed effects regression (DisMod-MR), a Bayesian meta-regression disease modeling tool; (ii) the non-specific codes of all available data on mortality were corrected and used to estimate mortality rates for ischemic stroke and the cause of death ensemble model was used to estimate mortality rates; and (iii) the disability-adjusted life years (DALYs) is the sum of years lived with disability (YLD) and the years of life lost (YLL), which quantifies the health loss due to specific diseases and injuries. In addition, the joinpoint regression model was adopted to analyze the temporal trend of ischemic stroke from 1990 to 2019 in Asia.

**Result:**

This study found an increase in the burden of ischemic stroke in 2019 compared to 1990. Moreover, the age-standardized incidence rate (ASIR) of ischemic stroke showed a gradual upward trend over the specific period. The age-standardized mortality rate (ASMR) showed a downward trend in Asia from 1990 to 2019. The burden of ischemic stroke was more concentrated on older age groups, particularly those older than 65 years. East Asia had the highest burden of ischemic stroke compared to other regions in Asia. Particularly, China, India, Indonesia, and Japan had the highest burdens of ischemic stroke among the Asian countries and regions. However, the population with the highest burden of ischemic stroke was still the elderly group.

**Conclusion:**

Based on our study, it is evident that the burden of ischemic stroke exists substantially and exhibits variations in the aspects of age, gender, and geographical region in Asia. Without targeted implementation of population-wide primary strategies for prevention and control, the burden of ischemic stroke is likely to worsen significantly in the future.

## 1 Introduction

Stroke is a group of acute cerebrovascular diseases caused by a sudden rupture of blood vessels in the brain or the inability of blood to flow into the brain due to vascular blockage, which has the characteristics of high morbidity, mortality, disability rate, and recurrence rate. In addition, stroke is the leading cause of disability in adults and the major cause of death worldwide (1–4). Based on the global burden of disease (GBD) 2019 survey, stroke was found to be one of the primary causes of disability-adjusted life years (DALYs) for people who were aged more than 50 years (5). Furthermore, stroke remains a significant global cause of death and disability and has a considerable economic impact concerning its treatment and post-stroke care, accounting for approximately 34% of global healthcare spending (6). Owing to the economic differences between Asia and the West, the incidence of stroke is higher in Asia compared to the West (7). In Asia, the overall incidence of stroke varies between 116/100,000 and 483/100,000 per year (8) and the economic burden of stroke is both severe and variable. A study in 2019 that compared the cost of stroke between Indonesia, Malaysia, and Singapore showed that the per-day care expenses were $135.55, $227.53, and $366.76, respectively, in these countries (7).

Ischemic stroke is characterized by the necrosis of brain tissue due to narrowing or occlusion of arteries that supply blood to the brain, resulting in insufficient blood supply to the brain, which has been reported to account for 80% of all stroke cases (9). The global burden of ischemic stroke had been reported previously (4, 10–12). There were 7,630,800 incident cases of ischemic stroke worldwide in 2019 based on the GBD study (13). The incidence of ischemic stroke showed a notable increase across the world, with a total increase of 87.55% from 1990 to 2019. During this 30 years period, deaths related to ischemic stroke increased by 60.68% and the number of DALYs experienced a corresponding increase of 56.72%.

Previous studies on the burden of ischemic stroke have primarily focused on a global level incidence, and a comprehensive analysis at the regional level, particularly in Asia, is lacking. This study aimed to evaluate and describe the burden of ischemic stroke systematically to bridge this gap in Asia by utilizing the GBD 2019 data and provide a significant scientific basis for the relevant departments to develop comprehensive prevention strategies and address the challenges posed by ischemic stroke.

## 2 Method

### 2.1 Case definition

The World Health Organization’s (WHO) clinical criteria define stroke as rapidly developing clinical signs indicating disturbance of cerebral function lasting more than 24 h (usually focal) or leading to death (14). Ischemic stroke is defined as an episode of neurological dysfunction caused by focal cerebral, spinal, or retinal infarction. The methods of GBD for determining the cause of death related to stroke and its subtypes have been previously described in the areas where neuroimaging is not available (15). The incidence of stroke is defined as the occurrence of first ever stroke based on a clinical diagnosis by a physician according to the WHO criteria. Ischemic strokes are defined as all atherosclerotic and thromboembolic events leading to compromised blood flow to brain tissue and subsequent infarction (2).

### 2.2 Data source

This study used data from the GBD 2019 study, which is available from the Institute for Health Metrics and Evaluation (IHME).^1^ As a continuous quality improvement, each annual GBD study re-estimated the entire time series. This process includes all known advances in data, modeling, estimation methods, and health knowledge, thereby ensuring that each GBD contained the most recent estimates (16). The GBD 2019 study, published in October 2020, performed epidemiological assessments of 286 causes of death, 369 diseases and injuries, and 87 risk factors from 204 countries and territories, with subnational assessments in some countries (16).

In this study, when we analyzed the specific countries and regions in Asia, we searched IHME with “Asia” as the search term and found a total of five regions: Central Asia, high-income Asia Pacific, South Asia, East Asia, and Southeast Asia. Among them, there are nine countries in Central Asia, four countries in the high-income Asia Pacific region, five countries in South Asia, two countries and a region in East Asia, and 13 countries in Southeast Asia.

### 2.3 Estimation framework

Methods for estimating the incidence of ischemic stroke, mortality, prevalence, and DALYs have been described previously (17, 18). The incidence of ischemic stroke was estimated using the disability model for the global burden of disease study-mixed effects regression (DisMod-MR), a Bayesian meta-regression disease modeling tool (19). The non-specific codes of all available data on mortality were corrected and used to estimate mortality rates for ischemic stroke (19). The cause of death ensemble model was used to estimate the mortality rates (19). The disability-adjusted life years (DALYs) is the sum of years lived with disability (YLD) and the years of life lost (YLL) (20), which quantifies the health loss due to specific diseases and injuries (16).

Age-standardized rates were based on the world standard population developed for the GBD study. GBD estimates for a disease burden are reported with a 95% uncertainty interval (UI), including the true value of a parameter with 95% probability (*P*). UI not only accounts for variance in parameter estimation but also uncertainty from data collection, model selection, and other sources of uncertainty under the parameter estimation process (16). More detailed information regarding the specific indices has been recommended in advanced journals or research publications (2).

### 2.4 Statistical description

In this study, we used R software (version 4.2.2), specifying “Asia” as the location and “ischemic stroke” as the chosen cause. We present changes in the age-standardized prevalence (ASP), ASMR, age-standardized disability-adjusted life years rate (ASDR), ASIR, number of cases, mortality number, DALYs, and the incidence from 1990 to 2019 to reflect the trends in the burden of ischemic stroke. This study also presents these indicators in Asia by country, region, gender, and age. Then, four countries with the highest number of cases were further identified and the gender and age distribution of the disease in these countries were analyzed separately.

### 2.5 Statistical analysis

We used Joinpoint regression software (version 4.9.1.0) to analyze the temporal trend from 1990 to 2019 in Asia. This statistical method helps to identify significant joinpoints, which are points where the trend changes significantly. The approach involves dividing the trend into multiple phases and calculating the annual percentage change (APC) and its 95% confidence intervals (CIs) for each phase. The average annual percentage change (AAPC) was then used to summarize the overall change in trends from 1990 to 2019. An increasing trend was indicated if the APC value was greater than zero, while a decreasing trend was indicated by a negative APC value. To determine whether a trend is statistically significant, we consider the *p*-value. If the *p*-value is lower than 0.05, we can conclude that the trend is significant, and conversely, if the *p*-value is greater than 0.05, then the trend is considered stable.

## 3 Results

### 3.1 Temporal distribution of ischemic stroke in Asia

As shown in Table 1, the ASIR of ischemic stroke in Asia increased from 96.09 per 100,000 population (95% UI, 81.73–113.82) in 1990 to 104.89 per 100,000 population (95% UI, 89.57–124.26) in 2019. The number of ischemic stroke cases increased from 1909736.10 (95% UI, 1623923.05–2272762.74) in 1990 to 4942470.01 (95% UI, 4183025.72–5841173.33) in 2019 in Asia. The mortality number of ischemic stroke increased from 834905.51 (95% UI, 746953.76–973377.56) in 1990 to 1980428.76 (95% UI, 1773797.86 to 2168207.70) in 2019 in Asia. Conversely, the ASMR of ischemic stroke decreased from 60.98 per 100,000 population (95% UI, 54.41 to 70.89) in 1990 to 48.69 per 100,000 population (95% UI, 43.48 to 53.37) in 2019 in Asia. The ASMR of ischemic stroke from 1990 to 2019 in men was higher than that in women; however, the ASIR from 1990 to 2019 in women was higher than that in men.

**Table 1:** Incidence and mortality of ischemic stroke in Asia, 1990 and 2019.

Year	Gender	Incidence		Mortality	
		Number of cases (95% UI)	ASIR (95% UI) per 100,000	Number of cases (95% UI)	ASMR (95% UI) per 100,000
1990	Male	887282.17 (754054.18 to 1062358.89)	91.54 (77.72 to 108.71)	425779.20 (371525.54 to 506959.73)	67.66 (59.85 to 79.72)
	Female	1022453.94 (870203.92 to 1218341.08)	99.88 (84.97 to 118.81)	409126.30 (353572.29 to 467284.12)	55.33 (47.60 to 63.26)
	Both	1909736.10 (1623923.05 to 2272762.74)	96.09 (81.73 to 113.82)	834905.51 (746953.76 to 973377.56)	60.98 (54.41 to 70.89)
2019	Male	2316510.75 (1954006.57 to 2734842.05)	102.37 (87.48 to 121.03)	1047346.38 (914252.60 to 1177458.71)	57.91 (50.85 to 64.64)
	Female	2625959.27 (2219068.13 to 3121676.37)	107.12 (91.05 to 127.35)	933082.38 (803995.28 to 1063035.09)	41.11 (35.31 to 46.93)
	Both	4942470.01 (4183025.72 to 5841173.33)	104.89 (89.57 to 124.26)	1980428.76 (1773797.86 to 2168207.70)	48.69 (43.48 to 53.37)

ASIR, age-standardized incidence rate; ASMR, age-standardized mortality rate; UI, uncertainty interval.

**Figure 1:**
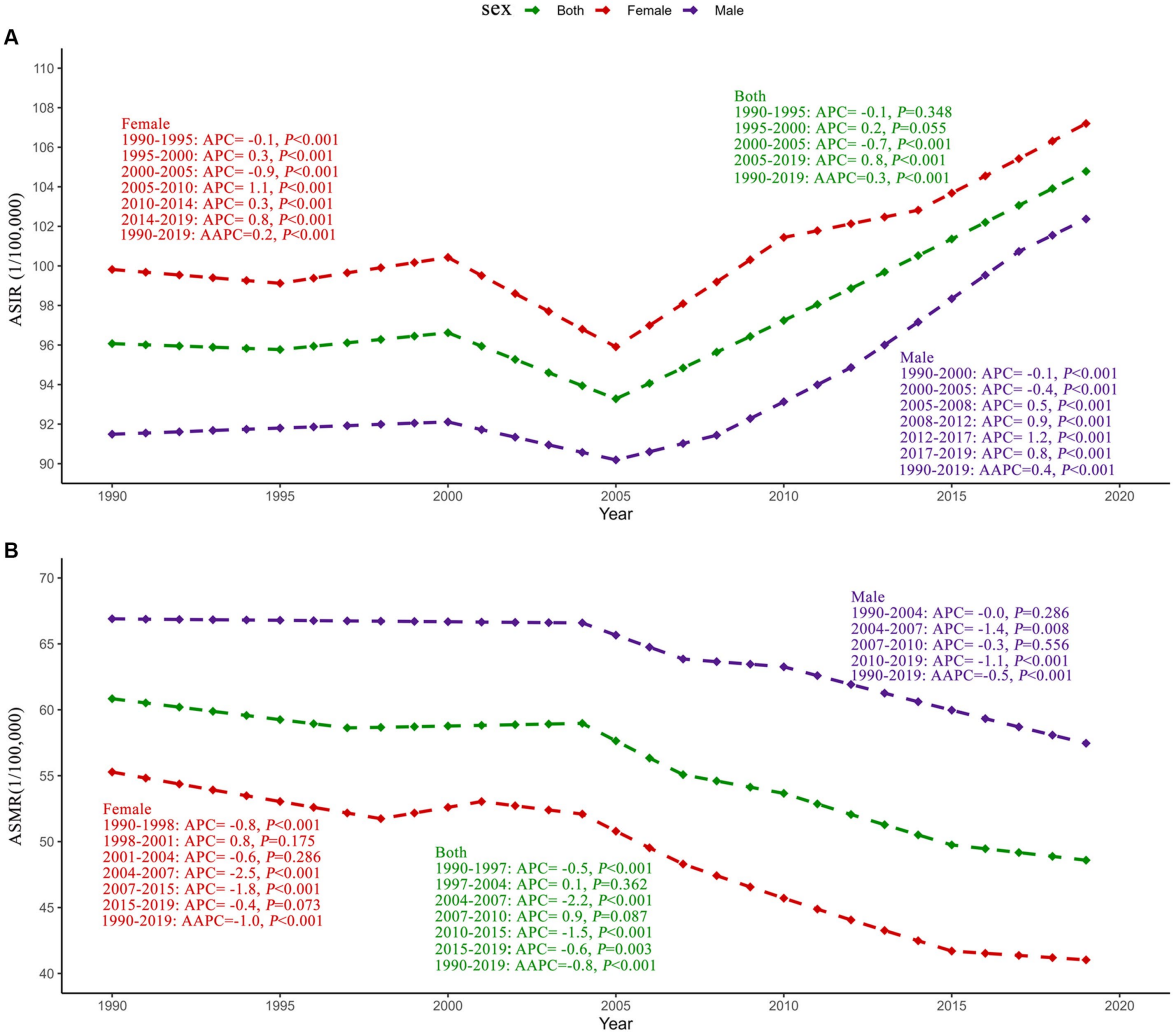
Joinpoint regression analysis of ischemic stroke ASIR **(A)** and ASMR **(B)** in Asia from 1990 to 2019. ASIR, age-standardized incidence rate; ASMR, age-standardized mortality rate; APC, annual percentage change; AAPC, average annual percentage change.

AAPC by Joinpoint regression analysis is shown in Figure 1. The ASIR of ischemic stroke in Asia showed an increasing trend from 1990 to 2019 (AAPC = 0.3, *p* < 0.001), and the most notable trends were observed between 2005 and 2019 (APC = 0.8, *p* < 0.001). Conversely, the ASMR of ischemic stroke exhibited an overall decreasing trend from 1990 to 2019 (AAPC = –0.8, *p* < 0.001), and the most notable declines were observed between 2004 and 2007 (APC = –2.2, *p* < 0.001) in Asia.

### 3.2 Population distribution of ischemic stroke in Asia

**Figure 2:**
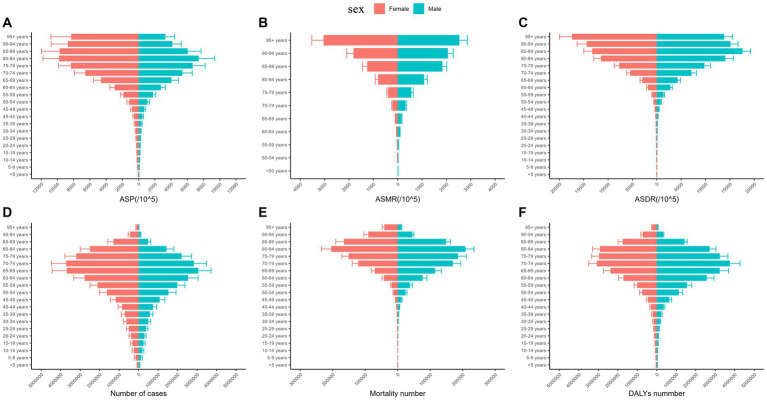
ASP **(A)**, ASMR **(B)**, ASDR **(C)**, number of cases **(D)**, mortality number **(E)**, and DALYs **(F)** due to ischemic stroke in Asia by gender in 2019. ASP, age-standardized prevalence; ASMR, age-standardized mortality rate; DALYs, disability-adjusted life years; ASDR, age-standardized disability-adjusted life years rate.

In terms of population distribution, the overall disease burden of ischemic stroke was concentrated in the elderly age group (>65 years) in 2019. Figure 2 shows that ASP, ASMR, and the ASDR of ischemic stroke increased with age, but their numbers showed unimodal distributions at different ages. A peak in the number of cases was observed at the age of 70–74 years for women and at the age of 65–69 years for men. The mortality rate of ischemic stroke peaks at the age of 80–84 years for both men and women. For both men and women, the peak age of DALYs due to ischemic stroke is 70–74 years. In 2019, the ASP and the number of cases of ischemic stroke in Asia in women were slightly higher than those in men, while the ASMR, ASDR, mortality number, and the DALYs due to ischemic stroke in women were lower than those in men.

### 3.3 Regional distribution of ischemic stroke in Asia

**Figure 3:**
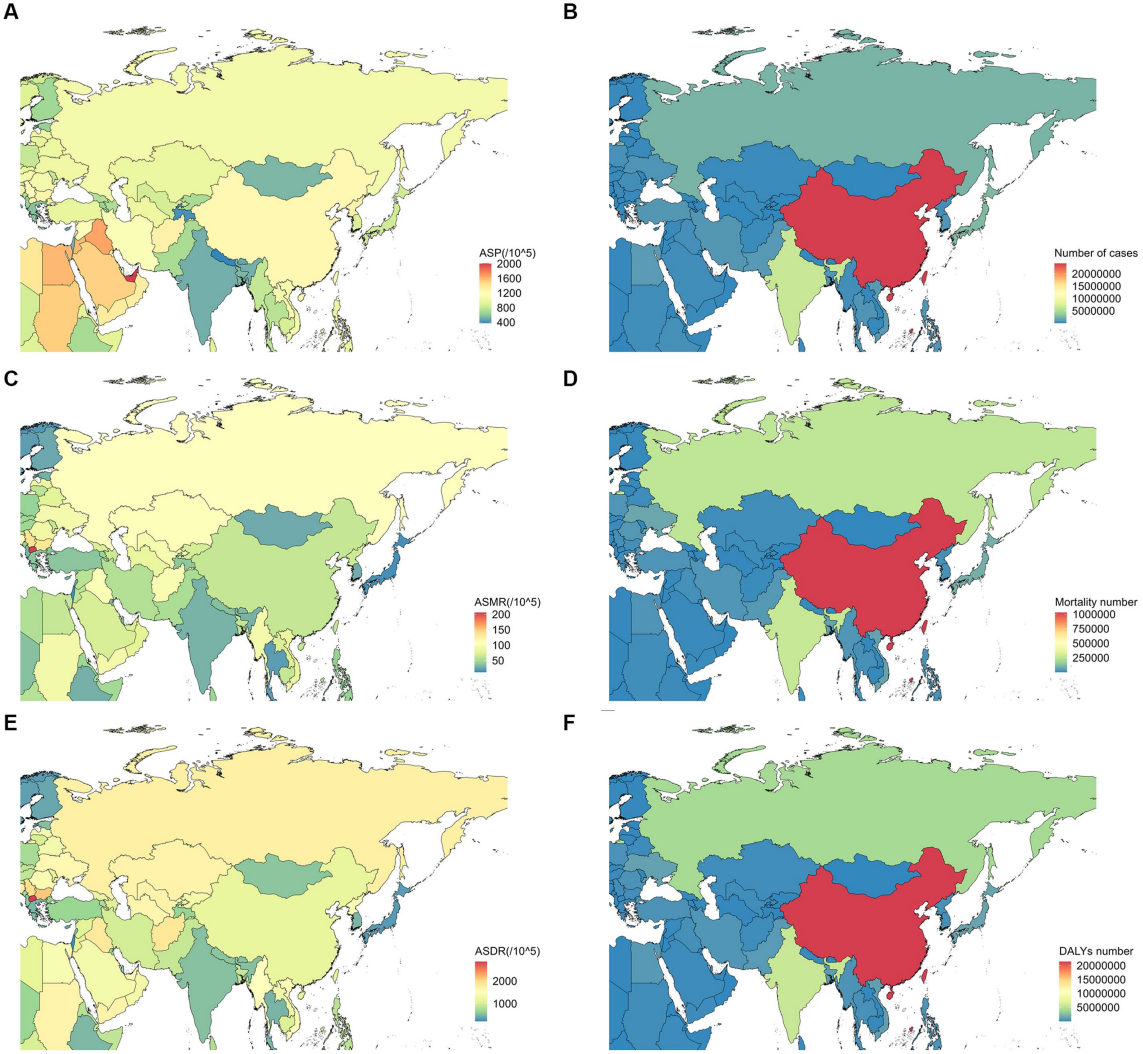
ASP **(A)**, number of cases **(B)**, ASMR **(C)**, mortality number **(D)**, ASDR **(E)**, and DALYs **(F)** due to ischemic stroke in different countries in Asia in 2019. ASP, age-standardized prevalence; ASMR, age-standardized mortality rate; DALYs, disability-adjusted life years; ASDR, age-standardized disability-adjusted life years rate.

At a regional level, the highest ASP (1249.23 per 100,000 population; 95% UI, 1081.19–1426.38), number of cases (24924020.48; 95% UI, 21474812.84–28619789.61), mortality number (1055199.34; 95% UI, 906555.40–1202264.51), and the DALYs (21980475.49; 95% UI, 19272756.33–24994300.25) of ischemic stroke were all in East Asia in 2019. The highest ASMR (79.43 per 100,000 population; 95% UI, 71.94–86.85) and ASDR (1386.79 per 100,000 population; 95% UI, 1269.80–1515.23) of ischemic stroke were noted in Central Asia (Table 2; Appendices 1, 2; Figure 3). The detailed burden of ischemic stroke by country in Asia is shown in Table 2 and Appendices 1, 2.

**Table 2:** Prevalence of ischemic stroke by country and region in Asia, 2019.

Location		Number of cases (95% UI)	ASP (95% UI) per 100,000
Asia		46833832.76 (41432208.34 to 53006529.39)	991.34 (876.10 to 1118.85)
East Asia	China	24183153.49 (20804402.52 to 27869456.30)	1255.91 (1083.57 to 1437.60)
	Democratic People’s Republic of Korea	408796.82 (368366.02 to 452265.38)	1302.51 (1171.88 to 1440.91)
	Taiwan (Province of China)	332070.17 (301585.31 to 363132.70)	894.86 (814.42 to 979.87)
	Total	24924020.48 (21474812.84 to 28619789.61)	1249.23 (1081.19 to 1426.38)
South Asia	Bangladesh	768324.77 (682372.32 to 860210.17)	545.61 (485.84 to 613.21)
	Bhutan	3112.30 (2784.97 to 3441.07)	492.71 (441.94 to 544.57)
	India	6465682.24 (5540992.14 to 7378042.38)	516.42 (443.55 to 590.04)
	Nepal	95029.39 (84927.71 to 105719.11)	376.70 (337.11 to 417.44)
	Pakistan	979624.12 (843707.55 to 1121097.12)	708.43 (610.77 to 815.30)
	Total	8311772.82 (7164911.02 to 9433858.71)	532.43 (461.49 to 606.48)
Central Asia	Armenia	26000.45 (23348.56 to 28904.46)	656.93 (589.98 to 727.77)
	Azerbaijan	72259.23 (64429.03 to 80019.13)	726.20 (650.35 to 805.26)
	Georgia	40094.20 (35020.42 to 46118.96)	735.51 (644.10 to 835.00)
	Kazakhstan	173563.47 (154399.06 to 194808.99)	972.80 (865.51 to 1093.25)
	Kyrgyzstan	35656.17 (31666.40 to 39543.87)	705.32 (627.78 to 779.62)
	Mongolia	14964.18 (13273.37 to 16663.08)	545.52 (486.52 to 607.29)
	Tajikistan	26938.64 (23769.45 to 30342.78)	413.34 (366.18 to 462.17)
	Turkmenistan	41397.52 (37639.69 to 45623.65)	982.25 (884.57 to 1088.56)
	Uzbekistan	210949.92 (185032.65 to 238507.52)	853.54 (746.55 to 972.74)
	Total	641823.78 (573075.72 to 712970.02)	806.82 (722.32 to 894.75)
Southeast Asia	Cambodia	105045.87 (95245.97 to 116640.49)	864.33 (783.79 to 960.18)
	Indonesia	3105437.78 (2658401.09 to 3616206.56)	1417.77 (1210.28 to 1667.91)
	Lao People’s Democratic Republic	47454.53 (42950.27 to 52290.82)	1044.05 (939.52 to 1165.68)
	Malaysia	296653.40 (270065.83 to 325983.16)	1086.82 (987.24 to 1197.15)
	Maldives	2611.53 (2360.43 to 2869.28)	759.25 (686.84 to 834.85)
	Mauritius	15084.10 (13748.74 to 16587.55)	910.33 (830.88 to 998.45)
	Myanmar	368739.56 (331904.28 to 406125.08)	788.43 (709.68 to 874.85)
	Philippines	780696.20 (678254.99 to 890430.69)	962.64 (834.73 to 1103.13)
	Seychelles	1165.82 (1062.53 to 1275.18)	1084.93 (986.37 to 1190.03)
	Sri Lanka	224553.36 (202351.91 to 250045.65)	904.23 (817.43 to 1009.10)
	Thailand	743350.32 (676390.65 to 818179.21)	759.36 (691.10 to 834.48)
	Timor-Leste	10017.21 (9051.92 to 11069.93)	1162.90 (1048.02 to 1291.37)
	Vietnam	975984.43 (883223.17 to 1082718.90)	1068.45 (960.01 to 1192.52)
	Total	6685552.86 (5898978.07 to 7569000.28)	1092.98 (963.48 to 1239.81)
High-income Asia Pacific	Japan	2678191.13 (2314694.63 to 3079537.07)	839.91 (737.73 to 948.45)
	Brunei Darussalam	3512.80 (3122.22 to 3991.16)	1160.67 (1034.00 to 1324.54)
	Republic of Korea	759142.28 (674522.38 to 872825.21)	884.37 (790.28 to 1009.59)
	Singapore	56421.40 (50888.09 to 62448.80)	740.17 (666.90 to 816.73)
	Total	3497267.61 (3061550.31 to 3990297.40)	848.74 (752.39 to 955.18)

ASP: age-standardized prevalence; UI: uncertainty interval.

**Figure 4:**
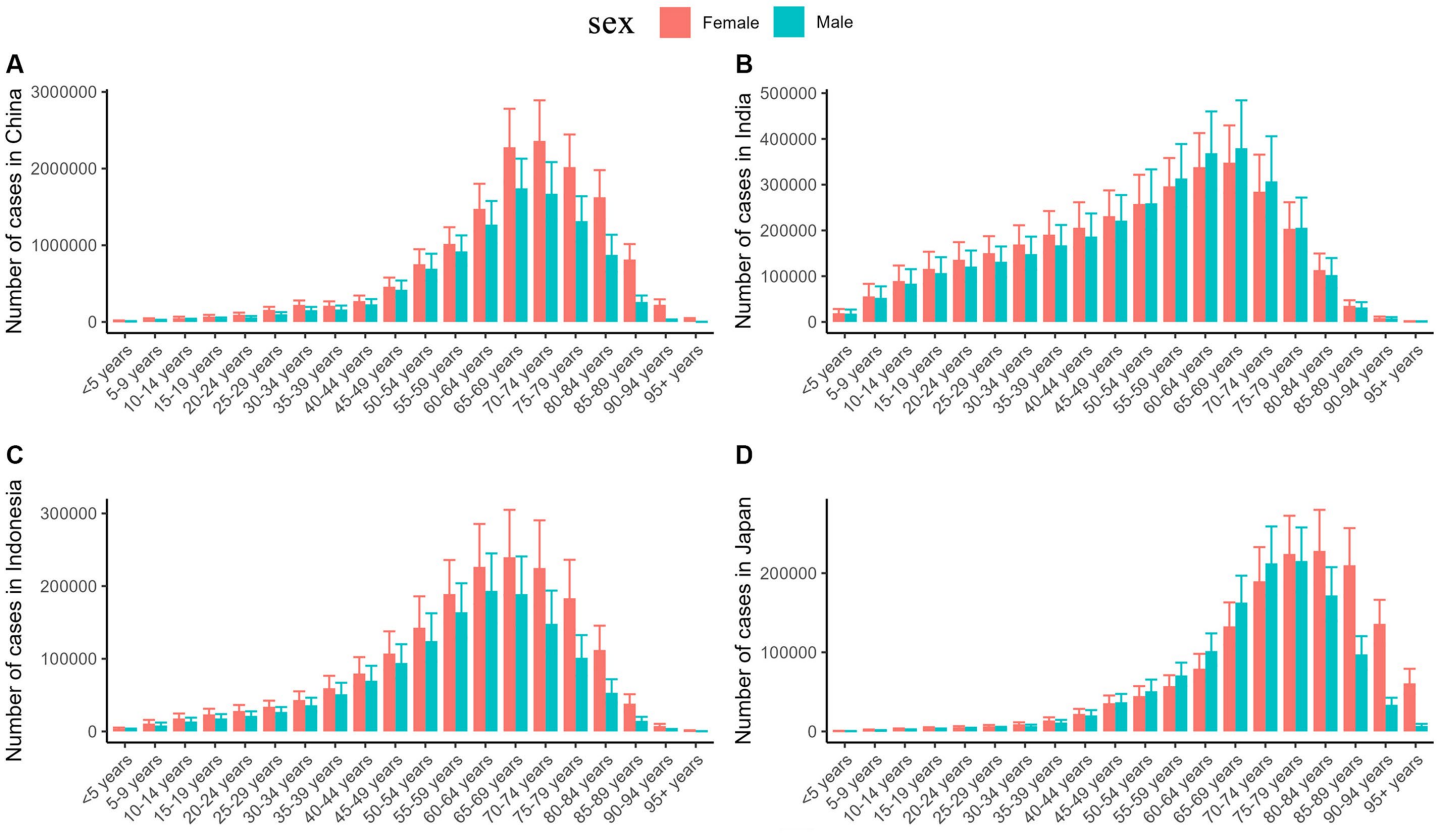
The number of ischemic stroke cases by age in China **(A)**, India **(B)**, Indonesia **(C)**, and Japan **(D)** in 2019.

Based on the above analysis, we selected four countries with the highest number of cases of ischemic stroke for further analysis: China (24183153.49; 95% UI, 20804402.52–27869456.30), India (6465682.24; 95% UI, 5540992.14–7378042.38), Indonesia (3105437.78; 95% UI, 2658401.09–3616206.56), and Japan (2678191.13; 95% UI, 1131346.60–1540307.05) (Table 2). The difference in the number of cases of ischemic stroke between different age groups almost had a unimodal distribution in different countries in 2019. In China, there was a peak in the incidence of ischemic stroke among women aged 70–74 years and men aged 65–69 years. In India, both men and women experienced a peak in the age group of 65–69 years. Indonesian women experienced a peak at the age of 65–69 years, while Indonesian men experienced a peak at the age of 60–64 years. Japan witnessed a peak in the number of cases of ischemic stroke among women aged 80–84 years and men aged 75–79 years. In China and Indonesia, the number of cases of ischemic stroke were higher in women than in men at all ages in 2019. However, in India, the number of cases of ischemic stroke was higher in women than in men before the age of 50 years and vice versa after the age of 50 years (Figure 4).

## 4 Discussion

This study found that the burden of ischemic stroke increased in Asia in 2019 compared to 1990. Moreover, the ASIR of ischemic stroke showed a gradual upward trend using Joinpoint regression analysis. However, this trend had decreased globally (13), which necessitates the focus of the WHO on the Asian region for preventing the incidence of stroke. Behavioral risk factors are the most important risk factors for ischemic stroke (21). Many countries and regions in Asia are in a period of economic development, and the consequent changes in eating habits and lifestyles have increased the exposure of their populations to risk factors for ischemic stroke despite increased awareness of medical approaches to stroke prevention and increased access to care. The ASIR of ischemic stroke had increased in Asia (22). In regions such as Asia, where the burden of stroke is high and increasing (23) and the rate of exposure to many important risk factors of stroke has been on the rise from 1990 to 2019 (24), current primary stroke prevention strategies and measures may not be sufficient and efforts to implement primary prevention strategies more widely in the population must be intensified, for example, strengthening health education and encouraging residents to develop a healthy lifestyle (25). The ASMR of ischemic stroke in Asia from 1990 to 2019 showed a downward trend. This result was consistent with the global findings (13, 26, 27). Even in high social development index (SDI) regions, the ASMR maintained a downward trend (13). The decrease in the ASMR in ischemic stroke may be attributed to the improvements of medical conditions and advancements in medical research. These advancements offer earlier detection of ischemic stroke-related risk factors and better control of these risk factors (28). Consequently, most of the patients have received effective treatment, resulting in a lower risk of death. Particularly, most high-income countries have established comprehensive monitoring systems for the risk factors of cardiovascular and cerebrovascular diseases (28). Therefore, the developing countries should pay more attention to the rational allocation of health resources, and the WHO should increase help to these countries to improve their medical technologies (22).

The ASIR from 1990 to 2019 in women was higher than that in men. However, the ASMR was higher in men than in women. The results are also consistent with global research (13). These findings suggest the possibility of an increased risk of disability and death caused by stroke in men and increased survival in women (29, 30). The variation in the exposure to risk factors for ischemic stroke between genders could account for this phenomenon. In 2019, the leading risk factors were high systolic blood pressure, environmental particulate pollutants, smoking, and a diet high in sodium for men. The leading risk factors were high systolic blood pressure, environmental particulate pollutants, high body mass index (BMI), and a diet high in sodium for women (31). Therefore, gender-specific interventions should be implemented for the effective control of ischemic stroke.

The study found that the burden of ischemic stroke was still concentrated in older age groups, particularly those older than 65 years, which was consistent with global research (13). Other national studies have also found similar results (32, 33). The current understanding of the mechanisms of ischemic brain injury includes an appreciation of multicellular interactions within the neurovascular unit (NVU), which may determine the evolution of blood-brain barrier (BBB) damage, neuronal cell death, glial reaction, and immune cell infiltration (34). Recent studies provide evidence suggesting that aging can exacerbate the damage and dysfunction of various components of the neurovascular unit (NVU), consequently accelerating the progression of brain injuries (35). The risk factors of ischemic stroke include atrial fibrillation, hypertension, hyperlipidemia, hyperhomocysteinemia, diabetes, smoking, lack of physical activity, unhealthy diet, abdominal obesity, and alcohol consumption (36). Patients with ischemic stroke often exhibit certain risk factors in the early stages, and the accumulation of these risk factors tends to increase with age, making the occurrence of the disease more likely at advanced ages. For the prevention and control of ischemic stroke, measures should be taken at the front end of the chain of causation to prevent the occurrence of stroke-related chronic diseases by modifying the risk factors. Such measures may include adopting a balanced diet, quitting smoking and alcohol, indulging in regular exercise, and other lifestyle modifications. These efforts are important to further prevent and control ischemic stroke.

In the Asian region, East Asia had the highest ASP, number of cases, mortality number, and DALYs of ischemic stroke. Previous studies have shown that East Asia has the highest prevalence (13). The high population of countries in East Asia, such as China and Japan, contributes to an increased exposure to certain stroke risk factors. These factors include elevated blood pressure, high BMI, smoking, and high fasting blood sugar. The results of this study demonstrated that the highest ASMR and ASDR of ischemic stroke were noted in Central Asia. These disparities are complex and influenced by numerous factors, including racial and ethnic differences in access to healthcare, equity, socioeconomic determinants of health (37), and the accompanying lifestyle choices (38).

Specifically analyzing the number of cases of ischemic stroke in each country and region in Asia, we found that China, India, Indonesia, and Japan had the highest incidence among other Asian countries. First and foremost, countries such as China and India have sizable populations, which contributes to a larger number of cases. Second, as countries have different demographics and levels of population aging in Asia, there are significant regional differences in the burden of cardiovascular and cerebrovascular diseases. As mentioned earlier, the burden of ischemic stroke mainly concentrates in the older age group, and the burden is expected to further increase as population aging intensifies. In 2019, there were 164.5 million Chinese citizens aged 65 years and older and 26 million aged 80 years or older (39). Therefore, it is not surprising that China has the highest number of cases. Moreover, variations in stroke burden might be correlated with the differential quality of preventative care of stroke, stroke rehabilitation, and risk factors of stroke across different regions (31). Our findings will help to guide prioritization and resource allocation in different parts of Asia, particularly in countries with a high stroke burden.

The four high-burden countries exhibit slight variations in the age groups that experienced the peak number of cases. The peak was observed among people aged 60–70 years in India and Indonesia, while the peak number of cases was noted in the 70–74 years age group in China, which was similar to a previous study (31). The age group with a concentrated incidence is higher (women: 80–84 years old; men: 75–79 years old) in Japan than that of the other three countries, which may be due to the fact that Japan ranks the highest globally in terms of longevity. The average life expectancy in Japan was 81.4 years for men and 87.5 years for women in 2019 (33). Consequently, the peak age group in Japan is higher. There are more women (6.1%) than men (3.5%) in Japan who are over 80 years old; therefore, the peak age is higher in women than in men. In addition, it may also be related to dietary habits. Prospective studies have reported that closer adherence to Japanese dietary guidelines is associated with lower risks of total mortality and mortality from cardiovascular diseases in Japan (40). However, the Japanese dietary model adopts a more balanced diet, which may also be one of the reasons for the higher peak age group in Japan. This result suggests that high-burden countries should pay attention to this phenomenon in time (the burden of ischemic stroke in a country and society tends to be heavier when the age group affected is younger). Previous studies have provided limited detailed systematic analysis of the number of cases in each age group. Hence, conducting a comprehensive analysis of several countries with high case numbers is crucial in order to identify the underlying causes of the elevated levels in this particular age group and to develop effective preventive measures for the future.

The limitations of the study should be acknowledged. The data used in this research are estimates from GBD 2019, and their accuracy depends on the quality of the underlying data. The collection and availability of ischemic stroke data may vary across regions, leading to underestimations of the burden in certain areas. Furthermore, this study only described the burden of ischemic stroke and did not explore the related risk factors, suggesting the need for additional research in this area.

## 5 Conclusion

Ischemic stroke poses significant threats to public health and economic development. According to our study, the disease burden of ischemic stroke is high and varies by age, gender, and region in Asia. Without targeted implementation of population-wide primary ischemic stroke prevention strategies, the burden of ischemic stroke is likely to worsen significantly. Therefore, corresponding and effective measures should be taken for different groups of people and different regions, with an emphasis on primary prevention strategies, which is the simplest and most economical way to deal with the burden of ischemic stroke.

## Data availability statement

Publicly available datasets were analyzed in this study. This data can be found here: Institute For Health Metrics and Evaluation (IHME), Global Health Data Exchange (GHDx), Global Burden of Disease Study 2019 (GBD 2019), https://ghdx.healthdata.org/gbd-2019.

## Author contributions

XZ: Writing – original draft. HL: Writing – review & editing. XC: Writing – original draft. ML: Writing – original draft. JXZ and XJ: Writing – review & editing.

## Footnotes

^1^http://ghdx.healthdata.org/gbdresults-tool
