# Supplementary material for: Analysis of ischemic stroke burden in Asia from 1990 to 2019: based on the global burden of disease 2019 data
Source: Front Neurol. 2023 Dec 22;14:1309931. doi: 10.3389/fneur.2023.1309931 (PMC10770854; doi:10.3389/fneur.2023.1309931)
Supplement: Supplementary file 1 [file Table_1.DOCX]

| **Appendix 1 Mortality of ischemic stroke by country and region in Asia, 2019** | | | | | | |
| --- | --- | --- | --- | --- | --- | --- |
| Location | | |  | Mortality number(95%UI) |  | ASMR(95%UI) per 100,000 |
| Asia | | |  | 1,980,428.76(1,773,797.86 to 2,168,207.70) |  | 48.69(43.48 to 53.37) |
| East Asia |  | China |  | 1,029,318.17(881,393.98 to 1,176,348.10) |  | 62.18(53.27 to 70.65) |
|  |  | Democratic People's Republic of Korea |  | 19,093.32(15,924.43 to 22,866.69) |  | 66.22(54.93 to 79.73) |
|  |  | Taiwan(Province of China) |  | 6,787.85(5,346.09 to 8,369.85) |  | 16.66(13.16 to 20.59) |
|  |  | Total |  | 1,055,199.34(906,555.40 to 1,202,264.51) |  | 61.03(52.42 to 69.14) |
| South Asia |  | Bangladesh |  | 61,482.43(45,247.73 to 79,276.50) |  | 60.01(45.04 to 76.34) |
|  |  | Bhutan |  | 151.07(118.18 to 189.53) |  | 33.98(26.56 to 42.16) |
|  |  | India |  | 271,240.48(227,811.45 to 320,725.39) |  | 30.56(25.74 to 35.84) |
|  |  | Nepal |  | 5,958.43(4,273.03 to 8,297.71) |  | 35.82(26.39 to 48.58) |
|  |  | Pakistan |  | 38,987.93(32,247.04 to 51,898.50) |  | 52.14(43.07 to 68.78) |
|  |  | Total |  | 377,820.35(321,485.90 to 443,287.65 ) |  | 35.23(29.96 to 40.94) |
| Central Asia |  | Armenia |  | 1,775.97(1,490.82 to 2,071.83) |  | 45.10(37.97 to 52.35) |
|  |  | Azerbaijan |  | 3,617.40(2,836.50 to 4,279.87) |  | 64.91(51.70 to 76.38) |
|  |  | Georgia |  | 4,866.56(4,034.81 to 5,700.74) |  | 71.72(59.77 to 83.95) |
|  |  | Kazakhstan |  | 13,554.71(11,764.76 to 15,426.92) |  | 105.06(91.25 to 119.26) |
|  |  | Kyrgyzstan |  | 2,521.51(2,214.03 to 2,851.15) |  | 69.12(60.84 to 77.98) |
|  |  | Mongolia |  | 433.23(337.90 to 566.23) |  | 27.26(21.53 to 35.14) |
|  |  | Tajikistan |  | 1,050.68(857.93 to 1,308.13) |  | 39.57(32.72 to 48.07) |
|  |  | Turkmenistan |  | 2,572.04(2,049.99 to 3,171.48) |  | 82.37(66.32 to 100.37) |
|  |  | Uzbekistan |  | 10,942.42(9,295.62 to 12,839.32) |  | 95.26(83.25 to 109.48) |
|  |  | Total |  | 41,334.51(37,635.20 to 45,233.83) |  | 79.43(71.94 to 86.85) |
| Southeast Asia |  | Cambodia |  | 5,121.09(4,116.36 to 6,105.38) |  | 62.63(50.64 to 74.49) |
|  |  | Indonesia |  | 136,176.28(106,337.01 to 160,307.31) |  | 96.33(76.61 to 111.14) |
|  |  | Lao People's Democratic Republic |  | 2,137.78(1,702.81 to 2,690.08) |  | 71.90(58.51 to 88.76) |
|  |  | Malaysia |  | 8,201.91(6,442.62 to 10,435.25) |  | 40.11(31.84 to 51.02) |
|  |  | Maldives |  | 67.69(55.41 to 80.92) |  | 29.66(24.20 to 35.66) |
|  |  | Mauritius |  | 562.06(459.36 to 666.10) |  | 37.56(30.78 to 44.34) |
|  |  | Myanmar |  | 32,091.22(26,897.60 to 37,477.51) |  | 93.11(78.18 to 107.94) |
|  |  | Philippines |  | 24,336.17(20,468.70 to 28,435.08) |  | 42.99(36.23 to 49.75) |
|  |  | Seychelles |  | 34.62(28.72 to 41.15) |  | 39.07(32.56 to 46.26) |
|  |  | Sri Lanka |  | 8,991.87(6,795.70 to 11,597.61) |  | 45.90(34.85 to 58.23) |
|  |  | Thailand |  | 20,242.22(15,221.92 to 26,414.55) |  | 20.86(15.69 to 27.20) |
|  |  | Timor-Leste |  | 424.65(309.62 to 612.21) |  | 72.68(54.65 to 102.47) |
|  |  | Viet Nam |  | 62,379.5(50,288.46 to 73,514.83) |  | 85.54(68.50 to 100.45) |
|  |  | Total |  | 301,161.62(257,706.51 to 336,635.18) |  | 65.16(56.18 to 72.43) |
| High-income Asia Pacific |  | Japan |  | 91,175.67(69,971.44 to 103,724.01) |  | 15.88(12.65 to 17.83) |
|  |  | Brunei Darussalam |  | 58.12(51.18 to 66.34) |  | 41.29(36.15 to 47.54) |
|  |  | Republic of Korea |  | 22,578.62(18,897.91 to 27,428.88) |  | 27.83(23.14 to 33.50) |
|  |  | Singapore |  | 890.47(730.16 to 1,003.18) |  | 12.99(10.58 to 14.69) |
|  |  | Total |  | 114,702.87(89,698.13 to 129,656.94) |  | 17.63(14.12 to 19.67) |
| ASMR: Age-standardized Mortality rate; UI: Uncertainty interval | | | | | | |

| **Appendix 2 The DALYs of ischemic stroke by country and region in Asia, 2019** | | | | | | |
| --- | --- | --- | --- | --- | --- | --- |
| Location | | |  | Number of cases |  | ASDR(/10^5) |
| Asia | | |  | 40,763,868.41(36,728,167.50 to 44,682,830.14) |  | 905.42(818.29 to 991.35) |
| East Asia |  | China |  | 21,393,856.76(18,720,952.02 to 24,375,890.76) |  | 1,147.93(1,008.58 to 1,302.75) |
|  |  | Democratic People's Republic of Korea |  | 431,189.86(363,599.69 to 506,658.85) |  | 1,387.08(1,172.42 to 1,626.65) |
|  |  | Taiwan(Province of China) |  | 155,428.88(128,706.76 to 183,906.90) |  | 402.36(332.66 to 475.89) |
|  |  | Total |  | 21,980,475.49(19,272,756.33 to 24,994,300.25) |  | 1,135.03(997.93 to 1,284.34) |
| South Asia |  | Bangladesh |  | 1,056,724.08(793,190.71 to 1,381,291.29) |  | 898.24(676.58 to 1,159.08) |
|  |  | Bhutan |  | 2,829.17(2,293.31 to 3,476.84) |  | 553.59(450.89 to 680.02) |
|  |  | India |  | 5,689,263.86(4,821,067.25 to 6,649,548.06) |  | 541.35(461.58 to 633.24) |
|  |  | Nepal |  | 114,408.32(83,374.98 to 159,404.29) |  | 576.33(424.39 to 792.11) |
|  |  | Pakistan |  | 882,644.67(743,169.48 to 1,132,980.22) |  | 908.84(764.36 to 1,174.74) |
|  |  | Total |  | 7,745,870.10(6,612,010.10 to 9,077,243.40) |  | 605.29(521.10 to 706.71) |
| Central Asia |  | Armenia |  | 31,329.31(27,048.05 to 36,175.96) |  | 774.00(669.57 to 891.51) |
|  |  | Azerbaijan |  | 76,935.29(63,353.39 to 90,322.05) |  | 1,047.83(857.53 to 1,221.01) |
|  |  | Georgia |  | 74,654.99(63,817.75 to 86,559.59) |  | 1,189.28(1,020.09 to 1,371.68) |
|  |  | Kazakhstan |  | 260,682.49(229,951.36 to 294,014.46) |  | 1,715.39(1,515.35 to 1,929.68) |
|  |  | Kyrgyzstan |  | 53,950.08(47,821.13 to 60,948.08) |  | 1,287.44(1,142.42 to 1,453.48) |
|  |  | Mongolia |  | 12,772.05(10,292.86 to 16,014.01) |  | 574.46(466.01 to 724.96) |
|  |  | Tajikistan |  | 24,033.08(19,642.43 to 30,096.57) |  | 660.61(547.69 to 805.16) |
|  |  | Turkmenistan |  | 60,520.24(48,085.02 to 74,942.07) |  | 1,671.89(1,342.24 to 2,048.76) |
|  |  | Uzbekistan |  | 289,083.66(246,588.91 to 338,016.95) |  | 1,704.81(1,478.48 to 1,957.03) |
|  |  | Total |  | 883,961.20(807,144.73 to 973,322.84) |  | 1,386.79(1,269.80 to 1,515.23) |
| Southeast Asia |  | Cambodia |  | 102,759.43(84,531.72 to 121,155.95) |  | 1,034.14(855.22 to 1,206.47) |
|  |  | Indonesia |  | 2,957,904.29(2,298,331.42 to 3,475,193.84) |  | 1,654.43(1,322.30 to 1,917.55) |
|  |  | Lao People's Democratic Republic |  | 46,822.22(37,699.46 to 58,746.85) |  | 1,267.48(1,029.35 to 1,568.04) |
|  |  | Malaysia |  | 198,476.72(162,121.50 to 241,254.34) |  | 811.48(666.68 to 988.67) |
|  |  | Maldives |  | 1,500.65(1,268.36 to 1,752.95) |  | 544.37(460.58 to 633.94) |
|  |  | Mauritius |  | 11,190.17(9,456.11 to 13,023.64) |  | 697.71(590.64 to 810.56) |
|  |  | Myanmar |  | 597,175.29(498,286.66 to 700,052.41) |  | 1,497.32(1,267.13 to 1,733.16) |
|  |  | Philippines |  | 587,348.20(491,511.70 to 680,487.60) |  | 843.51(714.13 to 967.94) |
|  |  | Seychelles |  | 775.67(656.53 to 912.94) |  | 775.54(661.03 to 905.17) |
|  |  | Sri Lanka |  | 177,959.98(140,903.13 to 223,337.33) |  | 780.18(616.53 to 971.00) |
|  |  | Thailand |  | 464,144.69(368,546.40 to 572,097.59) |  | 471.48(376.25 to 579.94) |
|  |  | Timor-Leste |  | 9,292.59(6,993.60 to 12,803.83) |  | 1,280.07(976.54 to 1,756.59) |
|  |  | Viet Nam |  | 1,163,673.14(948,468.40 to 1,373,609.91) |  | 1,433.65(1,172.72 to 1,675.18) |
|  |  | Total |  | 6,327,312.45(5,438,880.58 to 7,129,649.13) |  | 1,175.56(1,018.24 to 1,313.00) |
| High-income Asia Pacific |  | Japan |  | 1,360,713.92(1,131,346.60 to 1,540,307.05) |  | 326.57(276.64 to 373.41) |
|  |  | Brunei Darussalam |  | 1,577.47(1,371.19 to 1,811.27) |  | 735.44(652.96 to 835.92) |
|  |  | Republic of Korea |  | 413,614.70(360,533.95 to 480,454.38) |  | 482.92(417.58 to 556.91) |
|  |  | Singapore |  | 20,596.10(17,455.92 to 23,673.12) |  | 282.11(239.46 to 323.58) |
|  |  | Total |  | 1,796,502.20(1,518,306.31 to 2,028,424.08) |  | 352.82(303.27 to 401.33) |
| ASDR: Age-standardized Disability adjusted of life years rate; UI: Uncertainty interval | | | | | | |
